# Associations between Dietary Glycemic Index and Glycemic Load Values and Cardiometabolic Risk Factors in Adults: Findings from the China Health and Nutrition Survey

**DOI:** 10.3390/nu13010116

**Published:** 2020-12-30

**Authors:** Minjuan Li, Zhixin Cui, Shuangli Meng, Ting Li, Tong Kang, Qi Ye, Mengting Cao, Yuxin Bi, Huicui Meng

**Affiliations:** 1 School of Public Health (Shenzhen), Sun Yat-sen University, Shenzhen 518106, China; limj36@mail2.sysu.edu.cn (M.L.); cuizhx3@mail2.sysu.edu.cn (Z.C.); mengshl@mail2.sysu.edu.cn (S.M.); liting66@mail2.sysu.edu.cn (T.L.); kangt7@mail2.sysu.edu.cn (T.K.); yeqi5@mail2.sysu.edu.cn (Q.Y.); caomt@mail2.sysu.edu.cn (M.C.); biyx5@mail2.sysu.edu.cn (Y.B.); 2 Guangdong Provincial Key Laboratory of Food, Nutrition and Health, Guangzhou 510080, China; 3 Guangdong Engineering Technology Center of Nutrition Transformation, Guangzhou 510080, China

**Keywords:** glycemic index and glycemic load, cardiometabolic risk factor, dyslipidemia, hyperglycemia, hyperuricemia

## Abstract

Studies investigating the associations between dietary glycemic index (GI) and glycemic load (GL) values and cardiometabolic risk factors (CMRF) among Chinese populations are strikingly limited. To assess the associations between dietary GI and GL values and CMRF, including dyslipidemia, hyperglycemia, and hyperuricemia in Chinese adults, we extracted data of 7886 apparently healthy adults from the 2009 wave of the China Health and Nutrition Survey. Dietary GI and GL values were calculated using data collected from three consecutive 24 h dietary recalls. Fasting lipid, glucose, and uric acid concentrations were measured and CMRF were defined on the basis of established criteria. There were no significant associations between dietary GI values and CMRF, and analyzing the data by age, sex, body mass index (BMI), and region did not alter these results. Dietary GL values were positively associated with prevalence of hyperuricemia in all participants (Q4 compared with Q1: odds ratio (OR) = 1.46; 95% CI: 1.14, 1.87; *p*-trend = 0.0030) and prevalence of hypercholesterolemia in participants ≥ 60 years old (Q5 compared with Q1: OR = 1.72; 95% CI: 1.11, 2.68; *p*-trend < 0.0010). Higher dietary GL but not GI values were associated with increased prevalence of hyperuricemia in apparently healthy Chinese adults and hypercholesterolemia in older Chinese adults. Further studies are required to confirm the public health implication of these findings.

## 1. Introduction

Despite decades of efforts in improving cardiovascular medicine, cardiovascular disease and type 2 diabetes, collectively known as cardiometabolic disorders, remain the leading causes of death in China and worldwide [1,2]. Dyslipidemia and hyperglycemia are the major cardiometabolic risk factors (CMRF), and elevated concentration of serum uric acid or hyperuricemia has been increasingly recognized as an emerging risk factor for cardiovascular disorders [3]. These traditional and emerging CMRF are highly prevalent, and there is strong evidence this situation could be partially attributable to a low adherence to a healthy diet [4,5,6]. Although certain carbohydrate-rich foods, such as refined grains and added sugar, have been found to be positively associated with circulating concentrations of lipid, lipoprotein, glucose, and uric acid, evidence is still inconclusive on the associations between carbohydrate quality and CMRF [7].

Glycemic index (GI) measures dietary carbohydrate quality, and glycemic load (GL) is a measure of both quantity and quality of dietary carbohydrate [8]. Since they were first introduced, questions have been raised as to whether they should be incorporated into nutrition labeling and dietary guidelines in assisting food choices [9,10,11,12]. Associations between dietary GI and GL values and traditional CMRF have been investigated in a considerable body of work with equivocal results [13,14,15,16,17,18,19,20,21,22,23,24]. While some studies have reported that low GI and/or low GL diets are associated with dysregulation in serum lipid and lipoprotein [13,14,15,16,17] and fasting glucose [18,19], other studies have reported null association [20,21,22,23,24]. The controversies may be attributable to population-specific reasons, such as dietary intake habits, food sources and compositions of carbohydrates, and individual physiological factors, in addition to methodological issues related to dietary GI and GL calculations [10,25,26]. The associations between dietary GI and GL values and other CMRF such as uric acid remain elusive. Only one interventional study reported that a low carbohydrate low GI diet reduces plasma uric acid concentrations in overweight or obese adults [27].

Traditional dietary patterns in China include carbohydrate-rich and high-GI foods as staple foods, which may contribute to increased risk for cardiometabolic disorders [5]. However, studies investigating the associations between dietary GI and GL values and CMRF among Chinese populations are extremely scarce and the findings are heterogeneous [28,29,30]. Of the studies available, they have not factored potential effect modifiers such as age, sex, body mass index (BMI), and regional characteristics of participants into the analyses [28,29,30]. The lack of information spawns unsubstantiated claims as to whether GI and GL should be included in dietary guidance or nutrition labeling in assisting Chinese populations to make food choices.

The purpose of the present study was to assess the associations between dietary GI and GL values and CMRF, including dyslipidemia, hyperglycemia, and hyperuricemia, in a nationwide cohort of Chinese adults. Our hypothesis was that high GI and/or GL diets would be associated with higher prevalence of dyslipidemia, hyperglycemia, and hyperuricemia.

## 2. Materials and Methods

### 2.1. Study Population

We used data from the China Health and Nutrition Survey (CHNS), which is an ongoing prospective household-based study initiated in 1989. With the use of a multistage random cluster sampling design, CHNS selected samples from community-dwelling participants in 9 diverse provinces (including Liaoning, Jiangsu, Shandong, Hubei, Henan, Hunan, Guizhou, Guangxi, and Heilongjiang) between 1989 and 2011, and subjects from the 3 largest municipal cities (including Beijing, Shanghai, and Chongqing) were further added in 2011 [31,32]. The CHNS was approved by the Institutional Review Committees of the University of North Carolina at Chapel Hill and the National Institute of Nutrition and Health, the Chinese Center for Disease Control and Prevention [33,34], and written informed consent was obtained from all participants.

Data from adult participants (≥18 years old) who were enrolled in the 2009 wave of CHNS were extracted and analyzed in this study. In the current investigation, we excluded participants who had incomplete dietary records (*n* = 569), missing values in all biochemical measurements (*n* = 1420), or implausible intakes of total energy (<800 or >4200 kcal/day for men and <500 or >3500 kcal/day for women) (*n* = 117) [35]. In addition, participants who were diagnosed with myocardial infarction, stroke, apoplexy, cancer; had diabetes or were taking drugs known to affect lipid and glucose regulations (*n* = 427); or were women during pregnancy (*n* = 59) or breastfeeding (*n* = 42) were further excluded. The final analysis included 7886 participants, including 3690 males and 4196 females (see Figure S1 for participant flow).

### 2.2. Dietary Intake Data Collection and Assessment

Dietary intake information was collected with 3 consecutive 24 h dietary recalls (including 2 weekdays and 1 weekend) [36,37]. With assistance from qualified interviewers, participants provided information on the amount, type, preparation method, time, and location of each food item consumed over the past 24 h. A food weighing method at the household level was used to confirm the amount of food consumption over the same 3-day period [36,37]. The intakes of total energy and nutrients, including carbohydrate, protein, fat, dietary fiber, polyunsaturated fatty acids (PUFA), and saturated fatty acids (SFA), were calculated with the use of Chinese Food Composition Tables [38,39,40].

### 2.3. Calculations of Dietary GI and GL Values

Dietary GI and GL values were calculated on the basis of previously established methods [41,42,43]. The dietary GI values represent the overall quality of carbohydrate intake of the diet and were calculated by summing the GI contribution of each individual carbohydrate-containing food, which was obtained by multiplying the percentage of available carbohydrate in each food relative to the total available carbohydrate in the entire diet by the GI value of the specific food. The GI value of each carbohydrate-containing food was obtained from either Chinese Food Composition Table [38] or International Tables of Glycemic Index and Glycemic Load Values 2008 [42]. The dietary GL values reflect both quality and quantity of dietary carbohydrate intake and were calculated by multiplying the dietary GI value by the total amount of available carbohydrate in the total diet, divided by 100. The 3-day average dietary GI and GL values of each participant were used for the final analysis. All the nutrients and dietary GI and GL values were adjusted for total energy intake by using the residual method [44].

### 2.4. Assessment of Covariates

Data on the sociodemographic and lifestyle characteristics of participants, including education level, region, urbanization index, alcohol consumption, smoking status, and physical activity status, were collected with validated questionnaires under the instructions of trained interviewers [32].

Body weight and height of participants were measured with the use of calibrated equipment. Weight was measured to the nearest 0.1 kg, and height was measured to the nearest 0.1 cm. Body mass index (BMI) was calculated as weight (kg) divided by height squared (m^2^). Waist circumference was measured at the midpoint line between the lowest point of the ribs and the upper edge of the iliac crest using a tape to the nearest 0.1 cm. The hip circumference was obtained by measuring the circumference between the symphysis pubis and the most convex part of the gluteus maximus with a tape to the nearest 0.1 cm. Waist-to-hip ratio was calculated by dividing the waist circumference by the hip circumference. Blood pressure was measured with an automated blood pressure monitor 3 times after the participant had rested for at least 5 min in seated position, and there was at least 1 min interval during 2 measurements. The average of 3 independent blood pressure measurements was used in the analysis. Physical activity data were collected with a validated self-reported questionnaire, and the metabolic equivalent task hours per week (METs-h/week) for each participant were calculated on the basis of time and intensity of household, occupational, transportation, and leisure time activities [45].

### 2.5. Assessment of CMRF

Blood samples were drawn from each participant in the morning after an overnight fasting for 8–12 h. Blood samples were centrifuged at 3000× *g* for 15 min, and serum samples were collected immediately and stored at −86 °C for subsequent laboratory analysis. All samples were verified and analyzed in a national central laboratory in Beijing (Medical laboratory accreditation certificate: ISO 15189: 2007) according to strict quality control standards [46]. Serum total cholesterol (TC), high-density lipoprotein (HDL) cholesterol and low-density lipoprotein (LDL) cholesterol, triglyceride, glucose, and uric acid concentrations were measured on a Hitachi 7600 automated analyzer (Hitachi) with corresponding reagents (Randox Laboratories Ltd. for glucose and uric acid and Kyowa Medex Co. for others). Whole blood glycated hemoglobin A1c (HbA1c) levels were measured on an automatic clinical chemistry analyzer (model HLC-723G7; Tosoh). Serum insulin concentrations were measured by radioimmunoassay (North Institute of Bio-Tech), and the Homeostatic Model Assessment-Insulin Resistance (HOMA-IR) score was calculated with the Matthews formula [47]. Participants with dyslipidemia (hypercholesterolemia (total cholesterol ≥6.2 mmol/L), low HDL-cholesterol (HDL-cholesterol <1.0 mmol/L), elevated LDL-cholesterol (LDL-cholesterol ≥ 4.1 mmol/L), and hypertriglyceridemia (triglyceride ≥2.3 mmol/L)), hyperglycemia (fasting blood glucose ≥6.1 mmol/L), or hyperuricemia (fasting uric acid concentrations >420 μmol/L in males and >357 μmol/L in females) were identified on the basis of established criteria for Chinese populations [48,49,50].

### 2.6. Statistical Analysis

SAS for Windows (version 9.4; SAS Institute) was used for all statistical analyses. Participants were divided into 5 groups according to the quintiles (Q) of dietary GI or GL values. Correlations between dietary GI and GL values and dietary intakes of total energy, macronutrients, total fiber, SFA, and PUFA were analyzed via Spearman’s rank correlation. Logistic regression models were used to analyze the associations between dietary GI and GL values and CMRF, including dyslipidemia (hypercholesterolemia, low HDL-cholesterol, elevated LDL-cholesterol, and hypertriglyceridemia), hyperglycemia, and hyperuricemia. Model 1 was a univariable logistic regression model. Model 2 was a multivariable logistic regression model, and included various potential confounders, including age (<50 years, 50–54 years, 55–59 years, 60–64 years, or ≥65 years), sex (female or male), BMI (<18.5 kg/m^2^, 18.5–23.9 kg/m^2^, 24–27.9 kg/m^2^, or ≥28 kg/m^2^), urbanization index (low, medium, or high), physical activity status (METs-h/week, in tertiles), current smoker (yes or no), educational level (none or primary school, middle school, or high school and above), frequent alcohol consumption (yes or no), region (south (Jiangsu, Hubei, Hunan, Guizhou, and Guangxi provinces) or north (Liaoning, Heilongjiang, Shandong, and Henan provinces)), blood pressure (normal blood pressure (systolic blood pressure <120 and diastolic blood pressure <80 mm Hg), high-normal blood pressure (systolic blood pressure 120–139 and/or diastolic blood pressure 80–89 mm Hg), and hypertension (systolic blood pressure ≥140 and/or diastolic blood pressure ≥90 mm Hg)), total energy intake (kcal/day, in quintiles), total dietary fiber intake (g/d, in quintiles), and PUFA/SFA ratio (in quintiles). The percentage of energy from carbohydrate intake (<50, 50–65, or >65) was additionally adjusted as a potential confounder when the associations between dietary GI values and CMRF were analyzed. The lowest quintile of dietary GI or GL values were used as the reference group. Associations between total carbohydrate intake and CMRF were also analyzed with both models 1 and 2 without adjusting for the percentage of energy from carbohydrate intake in model 2. A test for linear trend was performed using dietary GI or GL values or total carbohydrate intake as continuous variables by assigning the median values of quintiles to the variables in the logistic regression model, and subsequent potential effect modification analyses were performed by age, gender, BMI, and region. Potential effect modification was tested for the associations between dietary GI or GL values and CMRF by age (18–59 years or ≥ 60 years), sex (female or male), BMI (<24 kg/m^2^ or ≥24 kg/m^2^), and region (north or south). Data of logistic regressions are presented as odds ratio (OR) and 95% confidence interval (95% CI). Linear regression models were used to analyze the associations between dietary GL values and insulin resistance markers, including fasting insulin and HbA1c concentrations, as well as HOMA-IR, in participants ≥60 years. Model 1 was a univariable linear regression model. Model 2 was a multivariable linear regression model, and also adjusted for the same potential confounders as in the multivariable logistic regression model. All statistical analyses were two-sided, and statistical significance was accepted at *p* < 0.05.

## 3. Results

### 3.1. Characteristics of Study Participants

Data from a total of 7886 participants were analyzed in this study. The participants had an average age of 50 ± 15 years, 53.2% were female, and all participants had an average BMI in the normal weight range. The average GI and GL values of all participants were 73 and 208, respectively. The average age, BMI, waist-to-hip ratio, and blood pressures of participants were similar across the quintiles of dietary GI or GL (Table 1). Participants with higher dietary GI or GL values were more likely to smoke and be physically active but were less likely to drink alcohol and to have attended high school and above than those who had lower dietary GI or GL values. Participants in the highest dietary GI or GL quintiles were more likely to have higher intakes of carbohydrate and to consume less total dietary fiber, fat, and protein than those in the lowest dietary GI or GL quintiles. The percentage of participants with dyslipidemia, hyperglycemia, or hyperuricemia ranged from 9.2% to 18.0%.

Significant correlations between dietary GI and GL values and dietary intakes of macronutrients, total fiber, SFA, and PUFA were observed in all participants, with Spearman’s rank correlation coefficients ranging from −0.86 (dietary GL values and fat intake) to 0.91 (dietary GL values and carbohydrate intake) (all *p* < 0.0001) (Table S1). Similar results were observed in participants both ≥60 years and <60 years.

### 3.2. The Associations between Dietary GI and GL Values and CMRF

In univariable logistic regression model, higher dietary GI values were significantly associated with higher prevalence for hypercholesterolemia, elevated LDL-cholesterol, and hyperuricemia (model 1; all *p*-trend < 0.05) (Table 2). However, in fully adjusted models, dietary GI values were not significantly associated with the prevalence of hypercholesterolemia, low HDL-cholesterol, elevated LDL-cholesterol, hypertriglyceridemia, hyperglycemia, or hyperuricemia (model 2; all *p*-trend > 0.05) (Table 2).

Higher dietary GL values were significantly associated with higher prevalence for hypercholesterolemia, elevated LDL-cholesterol, and hyperuricemia in univariable logistic regression models (model 1; all *p*-trend < 0.05) (Table 3). In fully adjusted models, higher dietary GL values were significantly associated with an increased prevalence of hyperuricemia (model 2; Q4 compared with Q1: OR = 1.46; 95% CI: 1.14, 1.87; *p*-trend = 0.0028) (Table 3). However, dietary GL values were not significantly associated with the prevalence of hypercholesterolemia, low HDL-cholesterol, elevated LDL-cholesterol, hypertriglyceridemia, or hyperglycemia in model 2 after adjusting for all potential confounders (model 2; all *p*-trend > 0.05) (Table 3).

The associations between total carbohydrate intake and CMRF were also assessed. In the univariable logistic regression model, higher total carbohydrate intake was significantly associated with higher prevalence for hypercholesterolemia, elevated LDL-cholesterol, hypertriglyceridemia, and hyperuricemia (model 1; all *p*-trend < 0.05) (Table S2). However, in fully adjusted models, there were no significant associations between carbohydrate intake and cardiometabolic risk factors (model 2; all *p*-trend ≥ 0.05) (Table S2).

### 3.3. Associations between Dietary GI and GL Values and CMRF Based on Potential Effect Modifiers

There was no evidence of effect modification of the associations between dietary GI values and hypercholesterolemia, low HDL-cholesterol, elevated LDL-cholesterol, hypertriglyceridemia, hyperglycemia, or hyperuricemia by age, sex, BMI, and region of participants (all *p*-interactions ≥ 0.05) (Table 4).

There was significant effect modification of the associations between dietary GL values and hypercholesterolemia by age (*p*-interaction = 0.0010) and low HDL-cholesterol by BMI (*p*-interaction = 0.0315), respectively (Table 5). Participants who were ≥60 years and had the highest quintiles of dietary GL values had a 72% higher prevalence of hypercholesterolemia in comparison to those had the lowest quintiles of GL values (Q5 compared with Q1: OR = 1.72; 95% CI: 1.11, 2.68; *p*-trend < 0.0010) (Table 5). In order to explore whether this relationship may be attributable to insulin resistance, we used multivariable linear regression models to assess the associations between fasting insulin and HbA1c levels and HOMA-IR scores in participants ≥60 years old. However, no significant associations between dietary GL values and insulin resistance markers were observed (Table S3). Dietary GL values were not significantly associated with hypercholesterolemia in participants < 60 years old (*p*-trend = 0.60) (Table 5). There was no significant effect modification of the associations between dietary GL values and hypercholesterolemia, low HDL-cholesterol, elevated LDL-cholesterol, hypertriglyceridemia, hyperglycemia, or hyperuricemia by sex or region of participants (all *p*-interaction > 0.05) (Table 5).

## 4. Discussion

Since the introduction of GI in 1981, numerous studies have been published on this topic [9,10,11,12]. Several studies and consortiums have been advocating the independent benefits of GI and GL in the prevention of cardiometabolic disorders via improving risk factors such as dyslipidemia and hyperglycemia [9,10,11,12]. However, studies investigating the associations between dietary GI and GL and CMRF demonstrated equivocal results [13,14,15,16,17,18,19,20,21,22,23,24], and data are strikingly limited for Chinese populations, who have habitual intake of high-GL diets and increasing cardiometabolic risk [28,29,30]. The current study was conducted to address this limitation via assessing the associations between dietary GI and GL values and CMRF, including dyslipidemia, hyperglycemia, and hyperuricemia, in a nationwide cohort of Chinese adults. The unique aspect of our work is that we investigated the associations between dietary GI and GL values and the prevalence of hyperuricemia, which has not been assessed in Chinese adults previously.

In our study, dietary GI and GL values were not significantly associated with the prevalence of dyslipidemia or hyperglycemia. These results were consistent with previous observational studies, which have found no significant associations between dietary GI or GL values and fasting serum concentrations of total cholesterol [14,18,51], HDL-cholesterol [21,24,51], LDL-cholesterol [16,51], triglyceride [24,51,52], or glucose [13,15,16,22,23,24]. In addition, several randomized controlled trials have reported no significant effect of low GI or GL diets on fasting serum total cholesterol, HDL-cholesterol, triglyceride, or glucose concentrations [20,53,54]. Despite these null findings, a prospective cohort study reported an inverse association between dietary GL values and fasting serum HDL-cholesterol concentrations in both type 2 diabetic patients and healthy adults in southeastern China [29]. A cross-sectional analysis of Chinese adults living in southwest China demonstrated positive associations between high dietary GI and GL values with higher fasting glucose concentrations and elevated risk of prediabetes [30]. In contrast, another study on participants living in northern China found positive associations between high dietary GL values and favorable concentrations of serum lipid and lipoproteins and lower prevalence of hypercholesterinemia and elevated LDL-cholesterol [28]. These studies indicate differential relationships between dietary GL values and lipid, lipoprotein, and glucose profiles between northern and southern Chinese adults. The discrepancies may partially result from the inter-individual variations following consumption of high GL diets, which may be attributable to the differences in regions and dietary patterns [55]. GL-mediated responses in insulin secretion and glycemic control may also be regulated via pathophysiological pathways, such as the opioid system, especially in obese individuals [56,57]. However, our study found no evidence of effect modification on the associations between dietary GI and GL values and prevalence of dyslipidemia or hyperglycemia by region.

In our study, higher dietary GL values were significantly associated with an increased prevalence of hyperuricemia. Despite little evidence being available on this topic, several studies have investigated the effect of dietary fiber on uric acid concentrations. Observational studies have reported inverse associations between dietary fiber intake and hyperuricemia risk in adults [58,59]. Randomized controlled trials have found that consumption of a fiber-rich diet or replacing a refined carbohydrate-enriched diet with a complex carbohydrate-enriched diet decreased serum concentrations of uric acid [60,61]. Data from animal studies suggest the suppressive effect of dietary fiber on uric acid concentration may be attributable, in part, to the ability of fiber to inhibit dietary purine or adenine absorption [62]. The positive associations between dietary GL values and prevalence of hyperuricemia may be partially due to the low intake of dietary fiber in participants, since we observed inverse correlations between dietary GL values and dietary fiber intake. Of note, the positive association between dietary GL values and prevalence of hyperuricemia was only observed in participants from southern rather than northern China. A recent study reported positive associations between traditional southern dietary patterns and dietary cadmium intake and increased prevalence of hyperuricemia in CHNS cohort [63]. Rice is the staple food and major source of carbohydrate in traditional southern dietary patterns, and may also be a potential source of dietary cadmium due to contamination [63,64]. Therefore, the positive association between dietary GL values and prevalence of hyperuricemia in southern participants may be attributed, in part, to the higher dietary cadmium intake from rice [63]. We also reported a lack of a significant association between dietary GI values and prevalence of hyperuricemia. In contrast to our finding, data from the OmniCarb Randomized Clinical Trial reported that lowering dietary GI values decreases plasma uric acid concentrations in overweight and obese adults [27]. However, observational and interventional studies exploring the relationship between intake of fructose, which is categorized as a low-GI food, and uric acid concentrations or hyperuricemia risk have generated mixed results [58,65]. While some studies have found that consumption of fructose-containing foods increases serum uric acid concentrations [65], other studies reported no associations [58]. The discrepancy between these previous studies and our study may be attributed, in part, to the differences in the race/ethnicity, BMI, and dietary background of study participants. Lacking data in this area, further interventional studies assessing the effect of high compared to low GI/GL diets on uric acid in Chinese adults are required.

In participants ≥ 60 years old, higher dietary GL values were associated with increased prevalence of hypercholesterolemia. One possible underlying mechanism is that consumption of high GL meals or diets results in postprandial hyperglycemia and increased release of counter-regulatory hormones and free fatty acids [66,67], which collectively contribute to beta-cell damage and insulin resistance in the long term and promote dyslipidemia [66,68,69,70] and cardiometabolic risk [71]. However, dietary GL values were not significantly associated with fasting glucose, insulin and HbA1c concentrations, and HOMA-IR in older adults in our study. We observed inverse correlations between dietary GL values and dietary fiber intake in older adults. Fiber-rich foods have been reported to reduce circulating cholesterol concentrations via suppressing cholesterol absorption and bile acid reabsorption, which have been partially attributable to either direct effect of fiber or indirect effect of gut microbiota and short-chain fatty acids [72,73,74]. Dietary GL values were not significantly associated with hypercholesterolemia in young and middle-aged adults, but the reasons for the age-specific differences remain to be determined.

On the basis of our and previous studies, we understand that dietary GI and GL values have sometimes been associated with CMRF, but not consistently. The inconsistency may be attributable, in part, to population-specific variations, such as race, genetic background, dietary patterns, and eating behaviors [13]. In addition, methodological issues related to dietary GI and GL may also contribute to the equivocal results [10,41,75,76,77]. By definition, dietary GI and GL values are calculated from GI values of individual carbohydrate-containing foods collected from dietary questionnaires. Concerns have been raised as to whether calculated meal or dietary GI and GL values are equal to accrual measured values [41,77]. It is also unclear whether dietary GI or GL values could be independently associated with cardiometabolic health because foods are consumed in clusters and dietary and food components consumed together with carbohydrate-containing foods may influence CMRF jointly and synergistically [78,79]. Furthermore, large intra-individual and inter-individual variabilities of GI value determination for a single food have been reported in a series of studies, and glycated hemoglobin concentrations and insulin index of individuals and protein-containing foods consumed together with or prior to the carbohydrate-containing foods cause the variability in GI value determinations [10,78,80]. In addition to the CMRF investigated in the current study, endothelial function also contributes to increased cardiometabolic risk, collectively with dyslipidemia and hyperglycemia [81]. Lacking data in this area, further studies investigating the associations between dietary GI and GL values and endothelial function are required [82]. Due to the regulatory effects of certain medications, such as metformin and statins, on glycemic response, insulin resistance, lipid metabolism, or endothelial function [83,84], whether the associations between dietary GI and GL values and CMRF may be influenced by medication use requires further investigation.

Our study has a number of strengths, including the large-scale coverage of Chinese residents from 11 provinces and the strict quality controls in collecting data on demographic characteristics, lifestyle behaviors, and biochemical biomarkers. Furthermore, the dietary intake of each participant was evaluated by using the 3-d 24-h dietary recall method combined with the food weighing method, which guaranteed the accuracy of the dietary intake data. Finally, potential effect modifiers were factored into the analyses to further explore the associations between dietary GI and GL values and CMRF. Our study also has a few limitations. The cross-sectional design made it impossible to establish causal relationships and explore underlying mechanisms between dietary GL values and prevalence of hyperuricemia in all participants and between dietary GL values and prevalence of hypercholesterolemia in participants ≥60 years old. In addition, although many confounders were adjusted for in the analyses to eliminate potential bias, there may have been other potential confounders that were not obvious to the authors.

## 5. Conclusions

In conclusion, dietary GI values were not associated with prevalence of dyslipidemia, hyperglycemia, or hyperuricemia either overall or by specific age, sex, BMI, and regional groups. Higher dietary GL values were associated with increased prevalence of hyperuricemia in all participants and were associated with increased prevalence of hypercholesterolemia in older adults. There is a lack of clinical trials that have investigated the effect of low versus high GI or GL diets on CMRF and cardiometabolic risk, and thus randomized controlled trials are needed to establish causal relationships. Further studies are also required to confirm the public health implications of our current findings in Chinese populations.

## Figures and Tables

**Table 1:** Sociodemographic, anthropometric, and lifestyle characteristics of 7886 Chinese adults who participated in the China Health and Nutrition Survey 2009 according to quintiles of dietary glycemic index (GI) and dietary glycemic load (GL) values (*N* = 7886) ^1^.

Variables	All	Quintiles of Dietary GI Values			Quintiles of Dietary GL Values		
	(*N* = 7886)	Q1 (*n* = 1550)	Q3 (*n* = 1589)	Q5 (*n* = 1578)	Q1 (*n* = 1574)	Q3 (*n* = 1576)	Q5 (*n* = 1582)
Median	-	64.2	75.2	80.1	148.7	210.3	264.5
Age, years	50 ± 15	50 ± 15	50 ± 15	50 ± 15	50 ± 15	50 ± 15	50 ± 15
Gender, *n* (%)							
Male	3690 (46.8)	654 (42.2)	755 (47.5)	772 (48.9)	716 (45.5)	752 (47.7)	760 (48.0)
Female	4196 (53.2)	896 (57.8)	834 (52.5)	806 (51.1)	858 (54.5)	824 (52.3)	822 (52.0)
BMI, kg/m^2^	23.3 ± 3.4	23.4 ± 3.4	23.4 ± 3.5	23.1 ± 3.5	23.5 ± 3.4	23.3 ± 3.4	23.1 ± 3.4
Waist-to-hip ratio	0.9 ± 0.1	0.9 ± 0.1	0.9 ± 0.1	0.9 ± 0.1	0.9 ± 0.1	0.9 ± 0.1	0.9 ± 0.1
Systolic blood pressure, mm Hg	124.3 ± 18.6	124.0 ± 18.9	124.1 ± 18.7	124.6 ± 19.1	123.9 ± 18.8	124.4 ± 18.7	124.3 ± 18.7
Diastolic blood pressure, mm Hg	80.4 ± 11.3	80.1 ± 11.2	80.7 ± 11.2	80.5 ± 11.6	80.0 ± 11.0	80.2 ± 11.4	81.1 ± 11.7
Urbanization index, *n* (%)							
Low	2623 (33.3)	264 (17.0)	505 (31.8)	797 (50.5)	215 (13.7)	428 (27.2)	1013 (64.0)
Medium	2600 (33.0)	526 (33.9)	575 (36.2)	478 (30.3)	470 (29.9)	620 (39.3)	409 (25.9)
High	2663 (33.7)	760 (49.1)	509 (32.0)	303 (19.2)	889 (56.4)	528 (33.5)	160 (10.1)
Region, *n* (%)							
North	3311 (42.0)	572 (36.9)	684 (43.0)	700 (44.4)	560 (35.6)	592 (37.6)	864 (54.6)
South	4575 (58.0)	978 (63.1)	905 (57.0)	878 (55.6)	1014 (64.4)	984 (62.4)	718 (45.4)
High school and above, *n* (%)	1901 (24.1)	526 (33.9)	371 (23.4)	227 (14.4)	572 (36.3)	376 (23.9)	189 (12.0)
Alcohol consumption, *n* (%)	2601 (33.0)	532 (34.3)	540 (34.0)	484 (30.7)	581 (36.9)	514 (32.6)	486 (30.7)
Current smoker, *n* (%)	2214 (28.1)	396 (25.6)	461 (29.0)	467 (29.6)	413 (26.2)	475 (30.1)	466 (29.5)
Physical activity, METs-h/week	69.0 ± 100.1	53.9 ± 88.2	70.4 ± 100.4	81.6 ± 107.8	44.7 ± 79.5	65.4 ± 94.3	99.2 ± 117.2
Total energy intake, kcal/day	1729.7 ± 14.5	1731.7 ± 19.8	1728.4 ± 13.5	1729.7 ± 9.9	1731.0 ± 25.5	1729.6 ± 10.0	1729.3 ± 6.8
Total dietary fiber intake, g/day	10.9 ± 5.3	13.6 ± 7.0	11.1 ± 5.1	8.5 ± 3.1	12.8 ± 7.5	10.6 ± 4.8	10.0 ± 3.4
PUFA/SFA ratio	0.7 ± 3.4	0.6 ± 0.8	0.6 ± 0.8	0.7 ± 1.1	0.6 ± 0.7	0.6 ± 0.6	1.2 ± 7.6
Carbohydrate intake, % energy	67.6 ± 10.5	61.4 ± 11.1	67.1 ± 9.2	73.8 ± 8.4	53.9 ± 7.9	67.7 ± 4.6	80.5 ± 3.8
Fat intake, % energy	18.0 ± 9.2	22.9 ± 9.5	18.4 ± 8.2	13.2 ± 7.9	29.2 ± 7.8	18.1 ± 4.7	7.1 ± 3.3
Protein intake, % energy	14.4 ± 3.0	15.7 ± 3.7	14.6 ± 2.7	13.0 ± 2.1	16.8 ± 3.6	14.2 ± 2.3	12.4 ± 1.6
Hypercholesterolemia, *n* (%)	726 (9.2)	169 (10.9)	148 (9.3)	133 (8.4)	172 (10.9)	151 (9.6)	119 (7.5)
Low HDL-cholesterol, *n* (%)	774 (9.9)	132 (8.5)	149 (9.4)	136 (8.6)	165 (10.5)	149 (9.5)	153 (9.7)
Elevated LDL-cholesterol, *n* (%)	858 (10.9)	191 (12.3)	182 (11.5)	158 (10.0)	199 (12.6)	175 (11.1)	143 (9.0)
Hypertriglyceridemia, *n* (%)	1414 (18.0)	282 (18.2)	313 (19.7)	232 (14.7)	294 (18.7)	290 (18.4)	253 (16.0)
Hyperglycemia, *n* (%)	931 (11.8)	188 (12.1)	202 (12.7)	174 (11.0)	189 (12.0)	196 (12.4)	161 (10.2)
Hyperuricemia, *n* (%)	1199 (15.2)	256 (16.6)	260 (16.4)	187 (11.9)	286 (18.2)	255 (16.2)	182 (11.5)

^1^ Data are presented as mean ± SD or *n* (%). GI, glycemic index; GL, glycemic load; METs, metabolic equivalent tasks; PUFA/SFA, polyunsaturated fatty acid to saturated fatty acid; Q, quintiles.

**Table 2:** The associations between quintiles of dietary GI values and cardiometabolic risk factors of 7886 Chinese adults who participated in the China Health and Nutrition Survey 2009 (*N* = 7886) ^1^.

Variables	Quintiles of Dietary GI Values					*p*-Trend ^2^
	Q1 (*n* = 1550)	Q2 (*n* = 1584)	Q3 (*n* = 1589)	Q4 (*n* = 1585)	Q5 (*n* = 1578)	
Range	<68.7	68.7–73.5	73.6–76.6	76.7–78.8	≥78.9	
Median	64.2	71.5	75.2	77.8	80.1	
Hypercholesterolemia						
Cases, *n*	169	144	148	132	133	
Model 1	1.00 (Ref)	1.23 (0.97, 1.55)	1.19 (0.94, 1.50)	1.35 (1.06, 1.72)	1.33 (1.05, 1.69)	0.0077
Model 2	1.00 (Ref)	1.23 (0.97, 1.57)	1.09 (0.85, 1.40)	1.11 (0.85, 1.46)	1.12 (0.84, 1.49)	0.46
Low HDL-cholesterol						
Cases, *n*	132	182	149	175	136	
Model 1	1.00 (Ref)	0.72 (0.57, 0.91)	0.90 (0.70, 1.15)	0.75 (0.60, 0.96)	0.99 (0.77, 1.27)	0.60
Model 2	1.00 (Ref)	0.72 (0.56, 0.92)	0.90 (0.69, 1.17)	0.77 (0.59, 1.01)	0.93 (0.69, 1.26)	0.41
Elevated LDL-cholesterol						
Cases, *n*	191	186	182	141	158	
Model 1	1.00 (Ref)	1.06 (0.85, 1.31)	1.08 (0.87, 1.35)	1.44 (1.15, 1.82)	1.26 (1.01, 1.58)	0.0037
Model 2	1.00 (Ref)	1.03 (0.82, 1.29)	0.96 (0.76, 1.21)	1.16 (0.89, 1.50)	1.04 (0.79, 1.36)	0.62
Hypertriglyceridemia						
Cases, *n*	282	281	313	306	232	
Model 1	1.00 (Ref)	1.03 (0.86, 1.24)	0.91 (0.76, 1.08)	0.93 (0.78, 1.11)	1.29 (1.07, 1.56)	0.22
Model 2	1.00 (Ref)	1.05 (0.86, 1.27)	0.88 (0.72, 1.07)	0.89 (0.73, 1.10)	1.20 (0.95, 1.51)	0.92
Hyperglycemia						
Cases, *n*	188	201	202	166	174	
Model 1	1.00 (Ref)	0.95 (0.77, 1.18)	0.95 (0.77, 1.17)	1.18 (0.95, 1.48)	1.12 (0.90, 1.39)	0.16
Model 2	1.00 (Ref)	0.98 (0.79, 1.23)	0.98 (0.78, 1.23)	1.18 (0.92, 1.51)	1.14 (0.88, 1.48)	0.26
Hyperuricemia						
Cases, *n*	256	254	260	242	187	
Model 1	1.00 (Ref)	1.01 (0.84, 1.22)	1.10 (0.91, 1.33)	1.47 (1.20, 1.81)	1.04 (0.86, 1.26)	0.0027
Model 2	1.00 (Ref)	0.97 (0.79, 1.20)	1.06 (0.85, 1.32)	1.35 (1.05, 1.73)	1.03 (0.84, 1.26)	0.13

^1^ Data are presented as odds rations (ORs) (95% CIs), which were calculated with the use of logistic regression models. GI, glycemic index; Q: quintiles; Ref, reference. Model 1 was a univariable logistic regression model. Model 2 was a multivariable logistic regression model and was adjusted for potential confounders, including age, sex, body mass index (BMI), urbanization index, physical activity status, smoking status, educational level, alcohol consumption, region, blood pressure, total energy intake, total dietary fiber intake, PUFA/SFA ratio, and percentage energy from carbohydrate intake. ^2^ Tests for linear trend were based on variables containing median values of each quintiles of dietary glycemic index.

**Table 3:** The associations between quintiles of dietary GL values and cardiometabolic risk factors of 7886 Chinese adults who participated in the China Health and Nutrition Survey 2009 (*N* = 7886) ^1^.

Variables	Quintiles of Dietary GL Values					*p*-Trend ^2^
	Q1 (*n* = 1574)	Q2 (*n* = 1577)	Q3 (*n* = 1576)	Q4 (*n* = 1577)	Q5 (*n* = 1582)	
Range	<170.0	170.0–198.0	198.1–221.8	221.9–247.3	≥247.4	
Median	148.7	185.4	210.3	233.7	264.5	
Hypercholesterolemia						
Cases, *n*	172	162	151	122	119	
Model 1	1.00 (Ref)	1.07 (0.86, 1.35)	1.16 (0.92, 1.46)	1.46 (1.15, 1.87)	1.51 (1.18, 1.93)	< 0.0010
Model 2	1.00 (Ref)	1.08 (0.85, 1.36)	1.14 (0.89, 1.46)	1.33 (1.00, 1.75)	1.25 (0.93, 1.68)	0.06
Low HDL-cholesterol						
Cases, *n*	165	143	149	164	153	
Model 1	1.00 (Ref)	1.18 (0.93, 1.49)	1.13 (0.89, 1.42)	1.02 (0.81, 1.28)	1.09 (0.87, 1.38)	0.81
Model 2	1.00 (Ref)	1.19 (0.93, 1.52)	1.13 (0.88, 1.45)	1.00 (0.77, 1.32)	1.08 (0.82, 1.44)	0.83
Elevated LDL-cholesterol						
Cases, *n*	199	201	175	140	143	
Model 1	1.00 (Ref)	0.99 (0.81, 1.23)	1.16 (0.94, 1.44)	1.49 (1.18, 1.87)	1.46 (1.16, 1.83)	< 0.0010
Model 2	1.00 (Ref)	0.98 (0.79, 1.22)	1.11 (0.88, 1.40)	1.31 (1.01, 1.71)	1.16 (0.88, 1.52)	0.10
Hypertriglyceridemia						
Cases, *n*	294	289	290	288	253	
Model 1	1.00 (Ref)	1.02 (0.85, 1.22)	1.02 (0.85, 1.22)	1.03 (0.86, 1.23)	1.21 (1.01, 1.46)	0.07
Model 2	1.00 (Ref)	0.96 (0.79, 1.16)	0.91 (0.74, 1.10)	0.84 (0.68, 1.04)	0.98 (0.78, 1.23)	0.53
Hyperglycemia						
Cases, *n*	189	198	196	187	161	
Model 1	1.00 (Ref)	0.95 (0.77, 1.18)	0.96 (0.78, 1.19)	1.02 (0.82, 1.26)	1.21 (0.97, 1.51)	0.10
Model 2	1.00 (Ref)	0.91 (0.73, 1.14)	0.90 (0.72, 1.14)	0.87 (0.68, 1.12)	0.95 (0.73, 1.25)	0.60
Hyperuricemia						
Cases, *n*	286	258	255	218	182	
Model 1	1.00 (Ref)	1.15 (0.96, 1.38)	1.39 (1.14, 1.68)	1.71 (1.40, 2.09)	1.14 (0.95, 1.37)	< 0.0010
Model 2	1.00 (Ref)	1.08 (0.88, 1.32)	1.26 (1.00, 1.58)	1.46 (1.14, 1.87)	1.10 (0.90, 1.34)	0.0028

^1^ Data are presented as ORs (95% CIs), which were calculated with the use of logistic regression models. GL, glycemic load; Q: quintiles; Ref, reference. Model 1 was a univariable logistic regression model. Model 2 was a multivariable logistic regression model, and adjusted for potential confounders, including age, sex, BMI, urbanization index, physical activity status, smoking status, educational level, alcohol consumption, region, blood pressure, total energy intake, total dietary fiber intake, and PUFA/SFA ratio. ^2^ Tests for linear trend were based on variable containing median values of each quintiles of dietary glycemic load.

**Table 4:** The associations between quintiles of dietary GI values and cardiometabolic risk factors of 7886 Chinese adults who participated in the China Health and Nutrition Survey 2009, stratified by age, sex, BMI, and region (*N* = 7886) ^1^.

Variables	Quintiles of Dietary GI Values					*p*-Trend ^2^	*p*-Interaction
	Q1 (*n* = 1550)	Q2 (*n* = 1584)	Q3 (*n* = 1589)	Q4 (*n* = 1585)	Q5 (*n* = 1578)		
Hypercholesterolemia							
Age ≥60	1.00 (Ref)	1.28 (0.87, 1.89)	1.29 (0.87, 1.91)	1.39 (0.90, 2.14)	1.64 (1.06, 2.55)	0.05	0.17
Age <60	1.00 (Ref)	1.20 (0.88, 1.63)	0.98 (0.72, 1.35)	0.98 (0.71, 1.36)	0.90 (0.64, 1.27)	0.94	
Female	1.00 (Ref)	1.11 (0.81, 1.51)	1.02 (0.74, 1.40)	0.99 (0.71, 1.39)	1.15 (0.80, 1.64)	0.33	0.60
Male	1.00 (Ref)	1.46 (0.99, 2.15)	1.21 (0.83, 1.77)	1.31 (0.88, 1.95)	1.10 (0.73, 1.64)	0.94	
BMI < 24 kg/m^2^	1.00 (Ref)	1.36 (0.96, 1.93)	1.43 (0.99, 2.06)	1.12 (0.78, 1.61)	1.08 (0.75, 1.56)	0.74	0.17
BMI ≥ 24 kg/m^2^	1.00 (Ref)	1.12 (0.80, 1.57)	0.87 (0.62, 1.22)	1.10 (0.76, 1.59)	1.18 (0.80, 1.75)	0.52	
North	1.00 (Ref)	1.55 (1.08, 2.24)	1.25 (0.87, 1.79)	1.17 (0.79, 1.72)	1.61 (1.06, 2.43)	0.46	0.11
South	1.00 (Ref)	1.03 (0.74, 1.42)	0.98 (0.70, 1.37)	1.07 (0.75, 1.52)	0.85 (0.60, 1.22)	0.71	
Low HDL-cholesterol							
Age ≥60	1.00 (Ref)	0.59 (0.35, 0.98)	0.80 (0.46, 1.38)	0.65 (0.38, 1.12)	0.91 (0.51, 1.61)	0.27	0.89
Age <60	1.00 (Ref)	0.76 (0.57, 1.01)	0.93 (0.69, 1.25)	0.81 (0.60, 1.09)	0.94 (0.68, 1.31)	0.64	
Female	1.00 (Ref)	0.77 (0.53, 1.14)	0.86 (0.58, 1.30)	0.68 (0.45, 1.01)	0.88 (0.57, 1.36)	0.87	0.72
Male	1.00 (Ref)	0.68 (0.49, 0.94)	0.92 (0.66, 1.29)	0.83 (0.59, 1.16)	0.96 (0.67, 1.39)	0.31	
BMI < 24 kg/m^2^	1.00 (Ref)	0.69 (0.48, 1.00)	0.95 (0.64, 1.41)	1.02 (0.68, 1.52)	1.02 (0.67, 1.54)	0.73	0.21
BMI ≥ 24 kg/m^2^	1.00 (Ref)	0.73 (0.53, 1.02)	0.86 (0.61, 1.21)	0.63 (0.45, 0.89)	0.87 (0.59, 1.27)	0.16	
North	1.00 (Ref)	0.81 (0.55, 1.19)	0.79 (0.54, 1.17)	0.73 (0.49, 1.08)	0.74 (0.49, 1.11)	0.10	0.05
South	1.00 (Ref)	0.64 (0.47, 0.89)	1.01 (0.71, 1.43)	0.80 (0.57, 1.14)	1.25 (0.83, 1.89)	0.59	
Elevated LDL-cholesterol							
Age ≥60	1.00 (Ref)	1.35 (0.93, 1.95)	1.20 (0.83, 1.73)	1.67 (1.10, 2.53)	1.35 (0.91, 2.01)	0.07	0.18
Age <60	1.00 (Ref)	0.88 (0.66, 1.17)	0.83 (0.62, 1.12)	0.94 (0.69, 1.29)	0.88 (0.64, 1.22)	0.82	
Female	1.00 (Ref)	0.97 (0.73, 1.29)	0.90 (0.66, 1.21)	1.13 (0.81, 1.57)	1.06 (0.76, 1.49)	0.73	0.87
Male	1.00 (Ref)	1.14 (0.80, 1.62)	1.05 (0.74, 1.50)	1.20 (0.83, 1.75)	1.01 (0.69, 1.48)	0.63	
BMI < 24 kg/m^2^	1.00 (Ref)	1.15 (0.84, 1.59)	1.07 (0.77, 1.48)	1.13 (0.80, 1.60)	0.96 (0.69, 1.35)	0.61	0.40
BMI ≥ 24 kg/m^2^	1.00 (Ref)	0.93 (0.68, 1.27)	0.86 (0.63, 1.19)	1.18 (0.83, 1.69)	1.15 (0.79, 1.67)	0.74	
North	1.00 (Ref)	1.36 (0.97, 1.92)	1.10 (0.78, 1.54)	1.17 (0.81, 1.70)	1.22 (0.83, 1.78)	0.94	0.21
South	1.00 (Ref)	0.84 (0.62, 1.13)	0.86 (0.63, 1.17)	1.15 (0.82, 1.61)	0.92 (0.65, 1.29)	0.38	
Hypertriglyceridemia							
Age ≥60	1.00 (Ref)	1.14 (0.79, 1.66)	1.05 (0.72, 1.53)	0.95 (0.65, 1.40)	1.31 (0.88, 1.96)	0.53	0.86
Age <60	1.00 (Ref)	1.02 (0.81, 1.27)	0.83 (0.66, 1.03)	0.87 (0.69, 1.10)	1.16 (0.90, 1.51)	0.87	
Female	1.00 (Ref)	1.04 (0.80, 1.36)	0.93 (0.71, 1.22)	0.98 (0.74, 1.30)	1.24 (0.92, 1.67)	0.60	0.85
Male	1.00 (Ref)	1.05 (0.80, 1.39)	0.83 (0.63, 1.09)	0.82 (0.62, 1.08)	1.16 (0.85, 1.58)	0.69	
BMI < 24 kg/m^2^	1.00 (Ref)	1.18 (0.89, 1.57)	1.04 (0.78, 1.38)	1.06 (0.79, 1.42)	1.52 (1.11, 2.08)	0.39	0.26
BMI ≥ 24 kg/m^2^	1.00 (Ref)	0.95 (0.73, 1.22)	0.76 (0.59, 0.99)	0.77 (0.59, 1.01)	0.99 (0.73, 1.32)	0.56	
North	1.00 (Ref)	1.19 (0.88, 1.61)	0.81 (0.61, 1.09)	0.95 (0.70, 1.29)	1.17 (0.85, 1.62)	0.90	0.35
South	1.00 (Ref)	0.95 (0.74, 1.22)	0.94 (0.73, 1.22)	0.86 (0.66, 1.11)	1.24 (0.93, 1.67)	0.85	
Hyperglycemia							
Age ≥60	1.00 (Ref)	0.80 (0.55, 1.16)	0.78 (0.54, 1.13)	1.04 (0.69, 1.55)	1.03 (0.69, 1.53)	0.87	0.58
Age <60	1.00 (Ref)	1.10 (0.84, 1.45)	1.11 (0.84, 1.47)	1.27 (0.94, 1.71)	1.21 (0.88, 1.65)	0.15	
Female	1.00 (Ref)	1.01 (0.73, 1.38)	0.85 (0.62, 1.16)	1.17 (0.82, 1.66)	1.14 (0.80, 1.62)	0.93	0.61
Male	1.00 (Ref)	0.96 (0.70, 1.31)	1.12 (0.81, 1.54)	1.19 (0.86, 1.66)	1.14 (0.81, 1.61)	0.14	
BMI < 24 kg/m^2^	1.00 (Ref)	1.25 (0.90, 1.74)	1.01 (0.73, 1.39)	1.36 (0.96, 1.93)	1.17 (0.84, 1.65)	0.30	0.25
BMI ≥ 24 kg/m^2^	1.00 (Ref)	0.80 (0.60, 1.09)	0.94 (0.69, 1.29)	1.04 (0.74, 1.44)	1.12 (0.78, 1.59)	0.55	
North	1.00 (Ref)	1.05 (0.74, 1.51)	1.01 (0.71, 1.44)	1.18 (0.80, 1.74)	0.97 (0.66, 1.41)	0.16	0.42
South	1.00 (Ref)	0.93 (0.70, 1.24)	0.95 (0.71, 1.27)	1.17 (0.86, 1.60)	1.31 (0.94, 1.83)	0.0277	
Hyperuricemia							
Age ≥60	1.00 (Ref)	1.07 (0.75, 1.53)	1.17 (0.81, 1.69)	1.15 (0.79, 1.68)	1.60 (1.08, 2.37)	0.09	0.71
Age <60	1.00 (Ref)	1.01 (0.79, 1.28)	0.90 (0.70, 1.15)	1.02 (0.79, 1.32)	1.25 (0.94, 1.66)	0.34	
Female	1.00 (Ref)	1.06 (0.79, 1.44)	1.02 (0.75, 1.38)	1.04 (0.75, 1.43)	1.42 (1.00, 2.01)	0.28	0.98
Male	1.00 (Ref)	1.00 (0.76, 1.31)	0.94 (0.72, 1.24)	1.06 (0.80, 1.41)	1.30 (0.96, 1.76)	0.23	
BMI < 24 kg/m^2^	1.00 (Ref)	1.02 (0.76, 1.35)	1.15 (0.86, 1.54)	1.18 (0.87, 1.60)	1.47 (1.06, 2.02)	0.10	0.42
BMI ≥ 24 kg/m^2^	1.00 (Ref)	1.02 (0.76, 1.35)	1.15 (0.86, 1.54)	1.18 (0.87, 1.60)	1.47 (1.06, 2.02)	0.61	
North	1.00 (Ref)	1.07 (0.76, 1.51)	1.01 (0.72, 1.44)	1.19 (0.82, 1.72)	1.40 (0.96, 2.05)	0.43	0.95
South	1.00 (Ref)	1.01 (0.79, 1.29)	0.96 (0.75, 1.23)	1.00 (0.77, 1.30)	1.33 (0.99, 1.79)	0.20	

^1^ Data are presented as ORs (95% CIs), which were calculated with the use of a multivariable logistic regression model. GI, glycemic index; Q: quintiles; Ref, reference. Potential confounders, including age, sex, BMI, urbanization index, physical activity status, smoking status, educational level, alcohol consumption, region, blood pressure, total energy intake, total dietary fiber intake, PUFA/SFA ratio, and percentage energy from carbohydrate intake were adjusted for in the model. ^2^ Tests for linear trend were based on variables containing median values of each quintiles of dietary glycemic index.

**Table 5:** The associations between quintiles of dietary GL values and cardiometabolic risk factors of 7886 Chinese adults who participated in the China Health and Nutrition Survey 2009, stratified by age, sex, BMI, and region (*N* = 7886) ^1^.

Variables	Quintiles of Dietary GL Values					*p*-Trend ^2^	*p*-Interaction
	Q1 (*n* = 1574)	Q2 (*n* = 1577)	Q3 (*n* = 1576)	Q4 (*n* = 1577)	Q5 (*n* = 1582)		
Hypercholesterolemia							
Age ≥60	1.00 (Ref)	1.03 (0.71, 1.48)	1.94 (1.28, 2.93)	1.94 (1.26, 2.99)	1.72 (1.11, 2.68)	< 0.0010	0.0010
Age <60	1.00 (Ref)	1.10 (0.81, 1.50)	0.85 (0.63, 1.15)	1.05 (0.75, 1.47)	1.02 (0.72, 1.45)	0.60	
Female	1.00 (Ref)	1.17 (0.86, 1.59)	1.16 (0.84, 1.60)	1.34 (0.94, 1.89)	1.18 (0.83, 1.68)	0.05	0.77
Male	1.00 (Ref)	0.96 (0.67, 1.38)	1.12 (0.77, 1.63)	1.32 (0.88, 1.99)	1.39 (0.90, 2.15)	0.32	
BMI < 24 kg/m^2^	1.00 (Ref)	1.08 (0.77, 1.52)	1.35 (0.94, 1.92)	1.44 (0.98, 2.11)	1.19 (0.81, 1.76)	0.24	0.52
BMI ≥ 24 kg/m^2^	1.00 (Ref)	1.07 (0.78, 1.49)	0.98 (0.70, 1.36)	1.23 (0.85, 1.77)	1.32 (0.90, 1.94)	0.17	
North	1.00 (Ref)	1.21 (0.86, 1.72)	1.74 (1.17, 2.57)	1.48 (1.00, 2.19)	1.55 (1.05, 2.30)	0.13	0.07
South	1.00 (Ref)	0.99 (0.72, 1.35)	0.87 (0.63, 1.19)	1.22 (0.85, 1.75)	1.04 (0.71, 1.53)	0.36	
Low HDL-cholesterol							
Age ≥60	1.00 (Ref)	0.97 (0.59, 1.61)	1.15 (0.68, 1.93)	0.90 (0.54, 1.49)	1.24 (0.71, 2.15)	0.62	0.68
Age <60	1.00 (Ref)	1.26 (0.95, 1.67)	1.12 (0.85, 1.49)	1.04 (0.77, 1.40)	1.05 (0.77, 1.44)	0.69	
Female	1.00 (Ref)	1.05 (0.70, 1.57)	0.99 (0.66, 1.50)	0.75 (0.50, 1.13)	0.77 (0.51, 1.16)	0.87	0.15
Male	1.00 (Ref)	1.28 (0.94, 1.74)	1.22 (0.89, 1.67)	1.21 (0.87, 1.68)	1.37 (0.97, 1.94)	0.63	
BMI < 24 kg/m^2^	1.00 (Ref)	0.93 (0.65, 1.35)	1.18 (0.80, 1.72)	1.13 (0.76, 1.67)	1.32 (0.88, 2.01)	0.30	0.0315
BMI ≥ 24 kg/m^2^	1.00 (Ref)	1.44 (1.04, 2.00)	1.10 (0.79, 1.52)	0.92 (0.66, 1.29)	0.94 (0.67, 1.33)	0.51	
North	1.00 (Ref)	1.21 (0.82, 1.78)	0.92 (0.63, 1.35)	0.81 (0.55, 1.19)	0.86 (0.59, 1.25)	0.09	0.16
South	1.00 (Ref)	1.16 (0.85, 1.59)	1.30 (0.94, 1.81)	1.18 (0.83, 1.66)	1.38 (0.93, 2.05)	0.0352	
Elevated LDL-cholesterol							
Age ≥60	1.00 (Ref)	1.02 (0.72, 1.45)	1.66 (1.12, 2.44)	1.60 (1.07, 2.39)	1.36 (0.90, 2.04)	0.0137	0.09
Age <60	1.00 (Ref)	0.95 (0.72, 1.25)	0.89 (0.67, 1.18)	1.16 (0.84, 1.60)	1.04 (0.75, 1.45)	0.52	
Female	1.00 (Ref)	1.07 (0.81, 1.43)	1.16 (0.86, 1.57)	1.38 (0.99, 1.91)	1.17 (0.83, 1.63)	0.06	0.89
Male	1.00 (Ref)	0.87 (0.62, 1.21)	1.04 (0.73, 1.47)	1.23 (0.84, 1.80)	1.15 (0.77, 1.71)	0.33	
BMI < 24 kg/m^2^	1.00 (Ref)	0.91 (0.67, 1.24)	1.08 (0.78, 1.50)	1.32 (0.92, 1.90)	0.97 (0.68, 1.39)	0.63	0.56
BMI ≥ 24 kg/m^2^	1.00 (Ref)	1.05 (0.78, 1.42)	1.12 (0.82, 1.54)	1.29 (0.92, 1.82)	1.38 (0.96, 1.98)	0.08	
North	1.00 (Ref)	1.18 (0.85, 1.66)	1.57 (1.08, 2.27)	1.34 (0.92, 1.94)	1.21 (0.85, 1.75)	0.62	0.09
South	1.00 (Ref)	0.86 (0.64, 1.14)	0.89 (0.66, 1.19)	1.29 (0.92, 1.81)	1.13 (0.79, 1.63)	0.06	
Hypertriglyceridemia							
Age ≥60	1.00 (Ref)	0.85 (0.59, 1.23)	1.10 (0.75, 1.61)	1.02 (0.69, 1.51)	0.90 (0.61, 1.33)	0.87	0.18
Age <60	1.00 (Ref)	1.00 (0.80, 1.25)	0.85 (0.68, 1.06)	0.79 (0.62, 1.00)	1.00 (0.78, 1.30)	0.47	
Female	1.00 (Ref)	1.21 (0.92, 1.59)	1.04 (0.79, 1.36)	0.92 (0.69, 1.23)	0.96 (0.72, 1.29)	0.89	0.07
Male	1.00 (Ref)	0.77 (0.59, 1.00)	0.80 (0.61, 1.05)	0.77 (0.58, 1.03)	1.00 (0.74, 1.36)	0.44	
BMI < 24 kg/m^2^	1.00 (Ref)	0.96 (0.72, 1.27)	1.02 (0.77, 1.36)	0.94 (0.69, 1.26)	1.25 (0.90, 1.72)	0.64	0.19
BMI ≥ 24 kg/m^2^	1.00 (Ref)	0.96 (0.75, 1.24)	0.83 (0.64, 1.07)	0.78 (0.59, 1.02)	0.82 (0.62, 1.08)	0.21	
North	1.00 (Ref)	0.93 (0.68, 1.26)	0.83 (0.61, 1.13)	0.71 (0.52, 0.97)	0.83 (0.61, 1.13)	0.0309	0.45
South	1.00 (Ref)	0.97 (0.76, 1.24)	0.95 (0.75, 1.22)	0.95 (0.73, 1.23)	1.12 (0.83, 1.51)	0.40	
Hyperglycemia							
Age ≥60	1.00 (Ref)	1.16 (0.80, 1.68)	0.98 (0.68, 1.41)	1.03 (0.70, 1.51)	0.97 (0.65, 1.43)	0.96	0.47
Age <60	1.00 (Ref)	0.80 (0.61, 1.06)	0.86 (0.64, 1.15)	0.79 (0.58, 1.07)	0.94 (0.68, 1.31)	0.62	
Female	1.00 (Ref)	0.87 (0.63, 1.20)	0.75 (0.54, 1.04)	0.84 (0.59, 1.19)	0.95 (0.66, 1.37)	0.28	0.46
Male	1.00 (Ref)	0.95 (0.70, 1.29)	1.08 (0.79, 1.48)	0.90 (0.65, 1.24)	0.95 (0.67, 1.34)	0.63	
BMI < 24 kg/m^2^	1.00 (Ref)	0.76 (0.55, 1.05)	0.97 (0.69, 1.37)	0.87 (0.61, 1.24)	0.77 (0.54, 1.11)	0.22	0.06
BMI ≥ 24 kg/m^2^	1.00 (Ref)	1.07 (0.79, 1.44)	0.84 (0.62, 1.14)	0.86 (0.62, 1.18)	1.16 (0.82, 1.65)	0.67	
North	1.00 (Ref)	0.93 (0.65, 1.33)	0.99 (0.68, 1.44)	0.82 (0.56, 1.19)	1.04 (0.71, 1.52)	0.18	0.76
South	1.00 (Ref)	0.90 (0.68, 1.20)	0.86 (0.64, 1.14)	0.91 (0.67, 1.24)	0.88 (0.62, 1.23)	0.93	
Hyperuricemia							
Age ≥60	1.00 (Ref)	1.04 (0.73, 1.48)	1.15 (0.80, 1.64)	1.22 (0.84, 1.77)	1.79 (1.19, 2.68)	0.0029	0.55
Age <60	1.00 (Ref)	1.12 (0.89, 1.42)	1.05 (0.82, 1.33)	1.27 (0.98, 1.66)	1.33 (1.01, 1.76)	0.06	
Female	1.00 (Ref)	1.25 (0.93, 1.68)	1.32 (0.97, 1.80)	1.40 (1.01, 1.94)	1.51 (1.08, 2.13)	0.0094	0.47
Male	1.00 (Ref)	1.00 (0.77, 1.29)	0.93 (0.72, 1.21)	1.16 (0.87, 1.55)	1.42 (1.04, 1.93)	0.0413	
BMI < 24 kg/m^2^	1.00 (Ref)	1.22 (0.92, 1.62)	1.17 (0.88, 1.55)	1.42 (1.05, 1.94)	1.74 (1.25, 2.42)	0.0029	0.59
BMI ≥ 24 kg/m^2^	1.00 (Ref)	1.00 (0.76, 1.30)	1.00 (0.76, 1.33)	1.12 (0.83, 1.52)	1.25 (0.91, 1.71)	0.15	
North	1.00 (Ref)	1.42 (0.99, 2.02)	0.97 (0.69, 1.36)	1.26 (0.88, 1.82)	1.49 (1.04, 2.13)	1.00	0.17
South	1.00 (Ref)	0.98 (0.77, 1.24)	1.14 (0.89, 1.46)	1.25 (0.96, 1.64)	1.45 (1.07, 1.95)	< 0.0010	

^1^ Data are presented as ORs (95% CIs), which were calculated with the use of a multivariable logistic regression model. GL, glycemic load; Q: quintiles; Ref, reference. Potential confounders, including age, sex, BMI, urbanization index, physical activity status, smoking status, educational level, alcohol consumption, region, blood pressure, total energy intake, total dietary fiber intake, and PUFA/SFA ratio were adjusted for in the model. ^2^ Tests for linear trend were based on variables containing median values of each quintiles of dietary glycemic load.

## Data Availability

Not applicable.

## Supplementary Materials

The following are available online at https://www.mdpi.com/2072-6643/13/1/116/s1: Table S1: Correlations between dietary GI and GL values and daily energy and nutrient intake of 7886 Chinese adults who participated in the China Health and Nutrition Survey 2009. Table S2. The associations between quintiles of dietary carbohydrate intake and cardiometabolic risk factors of 7886 Chinese adults who participated in the China Health and Nutrition Survey 2009. Table S3: Associations between dietary GL values and insulin resistance markers among participants (age ≥ 60 years old) of Chinese adults who participated in the China Health and Nutrition Survey 2009. Figure S1: Flow diagram of 7886 Chinese adults who participated in the China Health and Nutrition Survey 2009.
