# Supplementary material for: Associations between Dietary Glycemic Index and Glycemic Load Values and Cardiometabolic Risk Factors in Adults: Findings from the China Health and Nutrition Survey
Source: Nutrients. 2020 Dec 30;13(1):116. doi: 10.3390/nu13010116 (PMC7823666; doi:10.3390/nu13010116)

Supplemental Material

Supplemental Table S1. Correlations between dietary GI and GL values and daily energy and nutrients intake of 7886 Chinese adults who participated in China Health and Nutrition Survey 2009 [1].

Daily energy and nutrients intake	Dietary GI values		Dietary GL values	
	Spearman *r*	*P* values	Spearman *r*	*P* values
**All participants (*N* = 7886)**				
Total energy, kcal/d	-0.02	0.06	-0.01	0.50
Carbohydrate, % energy	0.43	< 0.0001	0.91	< 0.0001
Fat, % energy	-0.39	< 0.0001	-0.86	< 0.0001
Protein, % energy	-0.31	< 0.0001	-0.52	< 0.0001
Total dietary fiber, g/d	-0.35	< 0.0001	-0.13	< 0.0001
SFA, g/d	-0.34	< 0.0001	-0.79	< 0.0001
PUFA, g/d	-0.42	< 0.0001	-0.71	< 0.0001
**Participants ≥ 60 y (*n* = 2054)**				
Total energy, kcal/d	-0.07	0.0032	-0.17	< 0.0001
Carbohydrate, % energy	0.38	< 0.0001	0.89	< 0.0001
Fat, % energy	-0.34	< 0.0001	-0.85	< 0.0001
Protein, % energy	-0.29	< 0.0001	-0.51	< 0.0001
Total dietary fiber, g/d	-0.43	< 0.0001	-0.17	< 0.0001
SFA, g/d	-0.30	< 0.0001	-0.79	< 0.0001
PUFA, g/d	-0.38	< 0.0001	-0.71	< 0.0001
**Participants < 60 y (*n* = 5832)**				
Total energy, kcal/d	0	0.74	0.05	0.0002
Carbohydrate, % energy	0.45	< 0.0001	0.91	< 0.0001
Fat, % energy	-0.41	< 0.0001	-0.87	< 0.0001
Protein, % energy	-0.31	< 0.0001	-0.53	< 0.0001
Total dietary fiber, g/d	-0.32	< 0.0001	-0.11	< 0.0001
SFA, g/d	-0.35	< 0.0001	-0.80	< 0.0001
PUFA, g/d	-0.44	< 0.0001	-0.71	< 0.0001

[1] Correlations were determined via Spearman's rank correlation. GI, glycemic index; GL, glycemic load; PUFA, polyunsaturated fatty acid; SFA, saturated fatty acid.

Supplemental Material

Supplemental Table S2. The associations between quintiles of dietary total carbohydrate intake and cardiometabolic risk factors of 7886 Chinese adults who participated in China Health and Nutrition Survey 2009 [1].

Variables	Quintiles of Carbohydrate intake					*P*-trend [2]
	Q1 (n = 1574)	Q2 (n = 1577)	Q3 (n = 1577)	Q4 (n = 1580)	Q5 (n = 1578)	
Range, g/d	< 254.1	254.1 - 281.8	281.9 -306.1	306.2 - 333.9	≥ 334.0	
Median, g/d	232.70	269.10	293.90	318.55	350.75	
Hypercholesterolemia						
n	158	186	142	119	121	
Model 1	1.00 (Ref)	0.84 (0.67, 1.05)	1.13 (0.89, 1.43)	1.37 (1.07, 1.76)	1.35 (1.05, 1.73)	0.0002
Model 2	1.00 (Ref)	0.84 (0.66, 1.05)	1.10 (0.85, 1.42)	1.24 (0.93, 1.66)	1.13 (0.84, 1.51)	0.13
Low HDL-cholesterol						
n	162	157	136	154	165	
Model 1	1.00 (Ref)	1.05 (0.83, 1.33)	1.22 (0.96, 1.55)	1.07 (0.85, 1.35)	0.99 (0.78, 1.24)	0.98
Model 2	1.00 (Ref)	1.07 (0.84, 1.36)	1.29 (0.99, 1.67)	1.03 (0.78, 1.37)	0.96 (0.73, 1.27)	0.81
Elevated LDL-cholesterol						
n	196	209	166	143	144	
Model 1	1.00 (Ref)	0.94 (0.76, 1.15)	1.21 (0.97, 1.51)	1.43 (1.14, 1.80)	1.42 (1.13, 1.78)	<0.0001
Model 2	1.00 (Ref)	0.93 (0.75, 1.15)	1.17 (0.92, 1.48)	1.28 (0.98, 1.67)	1.14 (0.87, 1.50)	0.11
Hypertriglyceridemia						
n	301	295	291	257	270	
Model 1	1.00 (Ref)	1.03 (0.87, 1.24)	1.05 (0.88, 1.26)	1.22 (1.02, 1.47)	1.15 (0.96, 1.38)	0.0341
Model 2	1.00 (Ref)	0.99 (0.82, 1.19)	0.97 (0.80, 1.18)	1.00 (0.80, 1.24)	0.95 (0.76, 1.18)	0.67
Hyperglycemia						
n	194	202	189	174	172	
Model 1	1.00 (Ref)	0.96 (0.78, 1.19)	1.04 (0.84, 1.28)	1.14 (0.92, 1.42)	1.15 (0.93, 1.44)	0.08
Model 2	1.00 (Ref)	0.90 (0.73, 1.13)	0.96 (0.76, 1.21)	0.90 (0.70, 1.17)	0.89 (0.69, 1.16)	0.43
Hyperuricemia						
n	283	272	243	208	193	
Model 1	1.00 (Ref)	1.06 (0.88, 1.27)	1.21 (1.00, 1.46)	1.45 (1.19, 1.76)	1.58 (1.29, 1.92)	<0.0001

Supplemental Material

Model 2	1.00 (Ref)	0.96 (0.79, 1.17)	1.11 (0.90, 1.37)	1.15 (0.91, 1.45)	1.24 (0.97, 1.57)	0.05

[1] Data are presented as ORs (95% CIs), which were calculated with the use of logistic regression models. Q: quintiles; Ref, reference. Model 1 was a univariable logistic regression model. Model 2 was a multivariable logistic regression model, and adjusted for potential confounders, including age, sex, BMI, urbanization index, physical activity status, smoking status, educational level, alcohol consumption, region, blood pressure, total energy intake, total dietary fiber intake and PUFA: SFA ratio.

[2] Tests for linear trend were based on variable containing median values of each quintiles of dietary carbohydrate intake.

Supplemental Material

Supplemental Table S3. Associations between dietary GL values and insulin resistance markers among participants (age ≥ 60) of Chinese adults who participated in China Health and Nutrition Survey 2009 [1,2].

Insulin resistance markers	Dietary GL values		
	***β* coefficient**	**R^2**	***P* values**
Insulin, μIU/mL			
Model 1	-0.0009	0.4 %	0.0026
Model 2	-0.0001	10.7 %	0.83
HbA1c, %			
Model 1	-0.0001	0.1 %	0.15
Model 2	0	10.8 %	0.98
HOMA-IR			
Model 1	-0.0009	0.4 %	0.0025
Model 2	-0.0001	10.0%	0.87

[1] Data are presented as *β* coefficients per 1 unit of the dietary GL value. GL, glycemic load; HbA1c, glycated hemoglobin A1c; HOMA-IR, homeostasis model assessment-insulin resistance.

[2] Associations between the dietary GL values and insulin resistance markers were analyzed using linear regression models. Model 1 was a univariable linear regression model. Model 2 was a multivariable linear regression model, and adjusted for potential confounders, including age, sex, BMI, urbanization index, physical activity status, smoking status, educational level, alcohol consumption, region, blood pressure, total energy intake, total dietary fiber intake and PUFA: SFA ratio.

Supplemental Material

Supplemental Figure Captions.

Supplemental Figure S1. Flow diagram of 7886 Chinese adults who participated in China Health and Nutrition Survey 2009.

Supplemental Material

Supplemental Figure S1. Flow diagram of 7886 Chinese adults who participated in China Health and Nutrition Survey 2009.

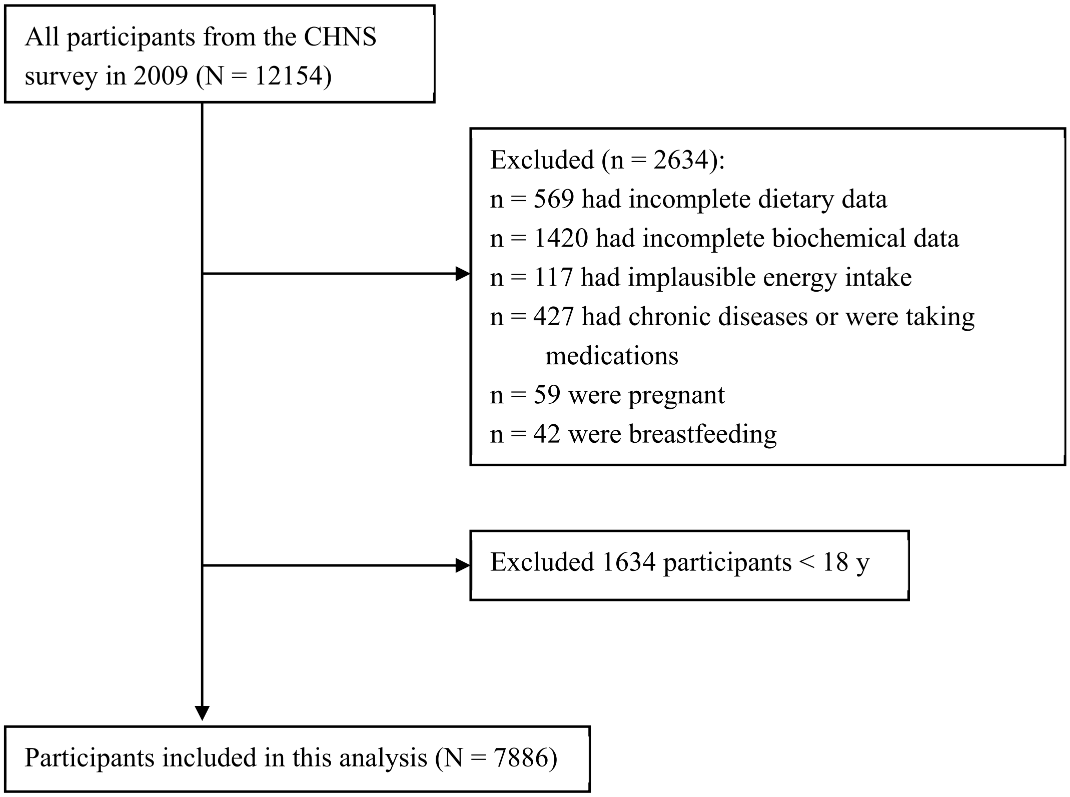

Supplement: Supplementary file 1 [file nutrients-13-00116-s001.pdf]
